# The Principles of Midwifery, Including the Diseases of Women and Children

**Published:** 1821-03-01

**Authors:** 


*The Principles of Midwifery; including the Diseases of Women and Children.* By John Burns, Regius Professor of Surgery in the University of Glasgow, &c. &c. Fifth and enlarged Edition. London, 1820.

The work which stands at the head of this article, has attained the honorable rank of a *fifth* edition—no inconsiderable proof of merit in a strictly professional publication. In glancing over an elementary work of this magnitude and standing, it can only be expected that we make a few cursory remarks on such practical points as attract our attention by the doubtful nature of their tendency, rather than their merits—for to particularize *these last* would require a space more than equal to the whole Journal.

We shall therefore confine ourselves to a review of the subject of Parturition which entirely occupies the second Book of the Volume.*

Dr. Burns, at page 341, describes the common method of examining for the purpose of ascertaining the existence and

---

* The following critical notice of Dr. Burns's work has been kindly furnished us by a Physician-Accoucheur of great talents and experience, and an author who is frequently quoted with approbation by Dr. Burns himself.—*Ed.*

progress of the labour, and adverts to the method proposed by Dr. Power, of trying a pain by examining the state of the uterus through the parietes of the abdomen. We need hardly tell our readers, that Dr. Power considers the application of the hand on the abdominal parietes, as affording a "very delicate and excellent mode of trying a pain; the information it gives is most important and uniformly correct; no genuine parturient action is without it; and when perfect, no false or unprofitable action is ever found co-existent with it, its presence evidencing the existence, and its absence the want of the true energetic internal principle."* It is always gratifying to find practitioners, under different circumstances, agreeing in opinion respecting such facts as may lead to practical improvement. We should have been pleased to find Dr. Burns's experience coinciding with that of Dr. Power, on this certainly, very delicate, and therefore desirable mode of trying a pain. But his expressions of approbation are by no means strong; all that he says upon the subject is, "the application of the hand to the abdomen during the continuance of the pain, may ascertain from the temporary hardness of the uterus, that its fibres are contracting universally. This mark has been properly insisted on by Mr. Power in his ingenious Treatise on Midwifery, p. 25" We seem to want further evidence on this important point of practice. For ourselves we can say, that either from a want of the true fact, or some other cause, we have not so uniformly obtained by this mode of examination the required information, as we had confidently expected, yet certainly on some occasions the knowledge thus acquired has been quite satisfactory.

In the chapter on Presentations of the superior Extremities, Dr. B. notices the spontaneous evolution of the Fœtus, and discusses the different theories of Drs. Denman, Douglas, and Kelly, respecting this curious phenomena, without attempting to decide between them. He concludes his observations by stating the following very important fact: viz. that in the city where he resides (Glasgow) the number of the inhabitants not being less than 150,000 one case only of the spontaneous evolution has been known to occur. Now as some women, with arm presentations, have died undelivered in that city, and as others have lost their lives, because delivery by turning was not effected till too late, it follows of course, that the spontaneous evolution is not to be depended upon. This then should operate as a caution against delay, and teach us

---

* Power's Treatise on Midwifery, developing new principles, &c.

not to wait, in vain hopes, that Nature will be competent to overcome the difficulty, but to proceed as early as practicable to effect delivery, by the old and well-tried method, turning the child, and extracting it by the feet.

The principal novelty in this edition is to be found in the chapter on *Tedious Labour*, on which Dr. Burns has written largely, and recommended the revival of a mode of practice gradually becoming, if not already become, obsolete.

It is well known, that the custom was formerly very prevalent of employing manual assistance to dilate the os uteri and vagina in all cases of slow labour, and very often in labours of a more active character. To so mischievous an extent, indeed, was this practice carried, that the teachers of midwifery, and the better informed practitioners, found it necessary to unite in inculcating the impropriety and danger of such proceeding, and more especially Dr. Hunter, whose reliance on the powers of Nature was almost unlimited, and to whose opinions great deference was paid, enjoined his pupils and others to leave the entire process of childbirth to Nature alone. The improvement which midwifery, as a science, derived from the doctrine thus promulgated, that Nature alone was equal to the power of effecting delivery in ordinary cases, was soon evident. It led to a more perfect and more extensive knowledge of the mechanism of parturition, and taught, that it was safe to trust to Nature, in some preternatural positions of the child, viz. in breech and feet presentations; nay, it produced still further good, by shewing, that in cases most urgently requiring artificial aid, Nature, if properly managed, was capable of doing a great deal, and that she ought, in all cases, to be considered as our firmest and most efficient ally.

It must, however, be admitted, that on some occasions this valuable rule of trusting to Nature has been perverted. By some, Nature, instead of being considered abundantly efficacious, has been thought all-sufficient; and in the absurd supposition that Nature could never fail, some accoucheurs have been found waiting, with dilatory, if not culpable, patience, for the natural termination of the labour, till the parturient woman has been exhausted with suffering, and the opportunity of doing good by the timely interference of art has been irretrievably lost.

The danger, thus to be feared from injudicious delay, has been foreseen, and all writers, even the most strenuous in deprecating premature interference, admit, that a time may arrive when the assistance of art must be given. Their rules respecting the period for interfering apply, however, only to labours of long duration; no modern author seems to have contemplated the propriety of employing artificial assistance during the first stage of labour. We have met with no book before this, which lays it down as a general rule, that the first stage of labour ought to be made to terminate within twelve hours of its commencement.

"In the case I have just considered, I have spoken of the effects of dilating the os uteri, but I do not mean to say, that the practice is useful in such a case alone; for, in most cases of tedious labour, it is beneficial, and, as the subject is important, I shall explain my sentiments on it fully. Forcible and irritating dilatation of the os uteri, even when it is not productive of dangerous consequences, is apt to occasion irregular or spasmodic action of the uterus. Two circumstances are necessary to render it safe: the os uteri must be lax and dilatable, and the dilatation must be gradually and gently effected during the continuance of a natural pain. If attempted in the absence of pain, and especially if attempted so as to give pain, it is apt to excite partial or spasmodic action, and under any circumstance, violent or forcible dilatation, besides injuring the uterine action, may lay the foundation of future disease. It is done best by pressing on the anterior edge of the os uteri, during a pain, with two fingers, with such moderate force as shall not give additional pain, and shall appear to excite the natural dilatation as much as to produce mechanical opening. By doing this for several pains in succession, or occasionally during a pain, at intervals, according to the effect produced and the disposition to yield, we shall soon have the os uteri completely dilated. This is an old principle, but it was rashly practised, and too universally adopted, which made it meet with just reprobation, and some, knowing this, may be surprised at meeting with such an advice in modern times. Let not the principle suffer from its abuse, else where is the plan which could stand its ground? It is perfectly clear, that when the process is going on well, interference is improper, but it is no less evident, that if a long time is to be spent in accomplishing the first stage of labour, or dilatation of the os uteri, the vigour of the uterus and strength of the patient may be impaired so much as to render the subsequent stage dangerously tedious, or to prevent its completion, at least consistently with safety; the first stage of labour ought always to be accomplished within a certain time, varying somewhat according to the constitution of the patient, and the degree of pain. It is an undeniable proposition, that there is in every case a period beyond which it cannot be protracted without exhaustion; and it is no less certain, that if we wish to avoid this exhaustion, which may be followed by pernicious effects, we have only the choice of either suspending the action altogether for a time, or of endeavouring to render it more efficient, and of effecting the desired object within a safe period. The first is sometimes adopted, but is not always practicable, nor is it always prudent to counteract uterine action by strong opiates. The second is safer, and one of the means of doing so is that under consideration. If the pain be continuing without suspension, or an interval of some hours, and the labour be going on all the time, but slowly, it is a good general rule to effect the dilatation of the os uteri within ten or twelve hours, at the farthest, from the commencement of regular labour. This is done, if the os uteri be flat and applied to the head, by the method above described. If it be somewhat projecting, it is aided by introducing two fingers, and extending them laterally with gentleness, during a pain. The dilatation is easily and safely effected, if the case be proper for it; if not, bleeding, or an opiate, if the former be not indicated, will soon bring about a favourable state. Of the benefit and perfect safety of this practice I can speak positively, and am happy to strengthen my position by the authority of Dr. Hamilton, who makes it a rule to have the first stage of labour finished within a given time. I need scarcely, however, add that, in enforcing this rule of conduct, it should be recollected that, to render it proper, the pains must be continuing so often and so decidedly, that the patient can be said to be in actual labour all the time. There are many cases where pains, at first regular, have gone off for many hours, or where they have come occasionally in a dull slight way, for a couple of days, but they have given little inconvenience, have scarcely interrupted sleep, and had little effect on the os uteri. They are more of the nature of false pains: the patient can hardly be said to be in labour, and is in no respect fatigued. If interference be proper in such cases, it is by other means, by opiates, by enemata, or remedies and applications evidently pointed out by the nature of the pains which have formerly been considered.

" If, again, in lingering labour, the membranes be entire, the os uteri soft, lax, and considerably dilated, and the presentation natural, it is allowable and beneficial to rupture the membranes; and this is more especially proper, if the uterus be unusually distended. The evacuation of the water is succeeded by more powerful action; a circumstance which, whilst it points out the advantage of the practice in the case under consideration, forbids its employment in natural labour, where the process is going on with a regularity and expedition, consistent with the views of nature, and the safety of the woman." *Burns on the Principles of Midwifery*, 401-2-3.

We must hesitate to call in question the propriety of a practice, recommended by an accoucheur of so much experience and judgment as Dr. Burns, and sanctioned by the authority of Dr. Hamilton; especially as Dr. Burns limits the practice, by admitting many exceptions to his general rule. Yet we cannot avoid remarking, that there is less of precision in the rule laid down than we admire.

The principle inculcated is, that the first stage of labour ought always to be accomplished within a certain time, which, we are afterwards told, ought not to exceed ten or twelve hours, from the commencement of the labour. But the term commencement of labour is very vague and indeterminate. By some the commencement is dated from the first appearance of discharge tinged with blood from the uterus:

but this is a very unsatisfactory evidence of labour. By others, the period of commencement is dated, at the occurrence of pains, but pains, spurious or preparatory, are often felt for hours and days before there is any, the slightest dilatation of the os uteri. Others again, refer the commencement of labour to the time when the os uteri begins to open: but a patent state of that orifice, even though accompanied with pains, does not always indicate the actuality of labour.*

How then, we ask, are we to know when labour commences? In what does it exist? Dr. Burns defines labour to be, "the expulsive effort made by the uterus for the birth of the child." Now our idea of an expulsive effort of the uterus is this, that first the fundus and body contract, and thus force the contents of the uterus towards the orifice, thereby pressing the os uteri lower into the vagina, and gradually dilating it, or rendering it thinner. The uterine effort then consists of a series of actions: viz. 1st. contraction; 2dly. propulsion; 3dly. depression, attenuation, or dilatation. If this be a correct definition of the expulsive effort of the uterus, or, in other words, of a labour pain, then, as soon as this series of actions commences, labour begins. But a finger in the vagina will frequently discover this expulsive effort before the os uteri is dilated to the size of a sixpence; and a continued succession of these well marked efforts may proceed regularly for many more than twelve hours, before the os uteri is open to the size of a shilling. It is clearly not Dr. Burns's intention to recommend, that the first stage of labour shall always be terminated within twelve hours from this period. Even then, if we were to admit the correctness of the principle, we think, that it is not explained with all that distinctness for which, in many other particulars, Dr. B. is entitled to deserved praise.

But our own experience does not warrant us to admit the correctness of the principle, or to receive this doctrine as a general rule. It seems to us, that the practice of dilating the os uteri is admissible only in a few cases, and applicable only after the labour has made a certain degree of progress. The stages of labour have been divided by some authors into three, by others into four periods. Dr. Burns prefers the first division, we are better pleased with the other; which exactly

---

* The first case in Chapman's Midwifery is strongly in point. "The mouth of the womb was fairly dilated, even enough to have admitted the hand;" but the pains were short and imperfect," that is to say, they were spurious, there was no uterine action. A composing draught and rest in bed relieved the pains, and the patient was not delivered till three weeks after.

divides Dr. B.'s first stage into a first and second. If the labour be thus divided, we would say, that in the cases supposed to require this kind of manual dilatation, the attempt ought not to be made, till the first stage is completed, nor then, except with very great caution; for it must never be forgotten, that the attempt to effect artificial dilatation, if not positively useful, is always positively hurtful.

If Dr. Burns differs from other writers respecting the management of the first stage, so does he in some degree, upon the use of instruments in the further progress of the labour.

The practitioners of midwifery of the last century seem to have been much too officious, in the use of the forceps, and other extracting instruments. To prove this, we have only to refer to the works of Chapman, Gifford, Burton, Smellie, &c. And there we may likewise learn, that in many of these instances the necessity of employing instruments arose out of the improper means resorted to, for the purpose of hurrying through the early period of the labour. The same authorities, which were the cause of altering the practice at the commencement of the labour, effected a change likewise as to the use of instruments towards its termination. From being too precipitate in affording artificial aid, it is probable that the accoucheurs of the present day may have fallen into the other extreme of being too tardy in giving such kind of assistance. Certain it is, that any instrumental interference has been decried in the most pertinacious manner, by some writers of great eminence. And the dread of employing instruments not only prevails in our own country, but on the Continent. Professor Boer, of Vienna, we are told in the Quarterly Journal of Foreign Medical Literature, "almost scoffs at instruments, and like Dr. William Hunter, sums up his advice for difficult cases in the word—patience." So Madame Boivin, midwife to the Hospice de la Maternité, at Paris, boasts of the infrequency of instrumental assistance, in that establishment, while she accuses the English practitioners of too great a fondness for such interference. Now, though it must be admitted, that it is essential to the character of a good accoucheur, not to be forward in interposing instrumental aid, yet the abstaining from instruments altogether cannot be the best criterion of his abilities. The preservation of the lives and of the future health and comfort of his patients is the paramount obligation of the accoucheur, and when these cannot be secured by patience, but may in all human probability by instruments, it becomes his duty not to delay their use.

These arguments have evidently been well considered by Dr. Burns. He says, p. 419,

" I have fully, and I hope practically detailed and considered the causes which render labour tedious, and have pointed out the impropriety of permitting the first stage to be protracted, for thereby the uterus becomes enfeebled, and less able to accomplish the second. But when this advice has not been acted on, or when the treatment proper for the particular cases already described, has not been successful in effecting delivery, what is the consequence ultimately of delay? The uterus, by continued, but inefficient action, or unavailing contraction, becomes gradually debilitated; and when at last delivery is effected, it cannot contract with vigour and regularity, whereby hæmorrhage is occasioned, or the same event is produced by spasmodic action of the uterus. Here then, is one very serious evil which may be anticipated. Next, there is a strong disposition given to puerperal disease, not merely to those troublesome, though less dangerous complaints, known under the name of weeds, or irregular febrile paroxisms; but also to more formidable effections, of an inflammatory nature, especially of the womb or peritoneum. Accordingly, we find that a much larger proportion of women die after protracted, than after natural, labour. Here then, is another class of evils to be apprehended. Again, although the same local mischief is not so apt to take place, that we meet with in locked head; yet, the patient is not exempted from risk even of that: by continuation of labour, the soft parts at last inflame and swell, which adds not only to the difficulty of delivery, but also greatly to the danger of the case. If it be necessary to enumerate other hazards, I may set down the consequence of protracted irritation and exertion, marked by the induction of a state of fever, and at last of great exhaustion, insomuch that the patient may actually die undelivered, but this event, as well as rupture of the uterus, is less apt to occur than in locked head. Besides all these hazards to the mother, the child is in danger of perishing, not from compression of the brain, but from the continued pressure of the uterus, after the evacuation of the water, interfering with the regular performance of the function of circulation. These are surely no trivial evils resulting from protracted labour; and the utmost that I feel at liberty to concede in favour of delay, is, that it may be permitted longer in cases of arrest, than of impaction. Many eminent men have placed an undue confidence in the power of nature, and have been hostile to the use of instruments. For a long time I was influenced by the high authority and plausible arguments, as well as bold assertions of these practitioners, but experience has compelled me to adopt the opinion I am now, with a firm and solemn belief of its correctness and importance, to maintain in this chapter. From the strength of the recommendations of the partizans of nature, we should suppose, that whenever the child could actually be born without aid, no hazard occurred, and, on the other hand, that instruments must of necessity prove not only very painful in their application, but dangerous in their effects. Now, the first supposition is notoriously wrong, for innumerable instances are met with, where the mother does bear her child, without artificial aid, and much, doubtless, to the temporary exultation of the practitioner, but nevertheless death takes place, or, at the best, a tedious and bad recovery is the consequence. The second supposition is just as positively unjust; for in the majority of cases, if the practitioner be humane and gentle, the introduction of the instrument gives little or no pain; in so much so, that in many books we meet with strong and just reprehension of the clandestine and unnecessary use of instruments, which could never possibly take place, if their application were attended in such cases with much pain. Then, as to the pain occasioned by extraction, that may be greater than the patient was just before suffering, and yet not be greater than is often experienced in a natural labour; or even granting it to be uniformly greater, a concession I make for the sake of argument, it is but for a short time, and, on the whole, the suffering of the patient is less than if nature had been allowed at length to expel the child. These positions are perfectly correct in all cases of arrest, when the practitioner is well instructed and cautious. Next, as to the danger to be apprehended, I cannot in cases of arrest see any source whence it can arise. The mere introduction of the forceps, if gently accomplished, can scarcely be more hazardous than the introduction of the finger, for no force is, or ought to be exerted. If there be hazard, it must be in the process of extraction, and this, it is evident, can arise only, either from pressure of the instrument on the soft parts, or from the head and instrument lacerating the perineum. The last event must, in general, be the consequence of want of caution, and the first can never be carried to any dangerous degree in a case of arrest, if the operator know how to direct his efforts." 420-1.

Dr. Burns afterwards enforces his arguments by referring to the tables published by Dr. Breen, calculated from the register of the Dublin Lying-in-Hospital.

"In the course of 57 years, 78,001 were delivered, of whom one out of every 92 died, and one child out of every 18 was stillborn. Let us now see the result of tedious labour.

In women, who were in labour of their first child from between 30 to 40 hours, one in 34 died, and one child in 5 was stillborn. Here then is a prodigious difference between even the average result of all labour, good and bad, and a protracted labour. During the same period of labour, amongst women who had previously borne children, and therefore, if requiring instruments, might be supposed to have a more permanent obstacle or contracted pelvis, though this is not stated, about one in every eleven died, and one child in every six was stillborn.

"When labour was protracted, between 40 and 50 hours, in women who had not previously borne children, one in 13 died, and the proportion of stillborn children was as one in $3\frac{1}{3}$.

"If labour were protracted other ten hours, that is, between 50 and 60, one-eleventh of the women died, and when we proceed to the period of between 60 and 70 hours, one eighth died, and nearly one-half of the children. It is observable however, that only onetwelfth died in the next ten hours, but this variation must arise from accidental circumstances.

"It is impossible to give any comparison of these results, with those afforded in the same hospital by the use of instruments, for artificial aid, it is evident, was always long delayed, unless in cases where dangerous symptoms not essential to labour occurred. Instruments were used, on account of tedious labour, in 44 cases, and of these 18 died.

Now, taking the proportion of deaths in the parturient state, to be, including all disasters whatever, as 1 in 92, it is most important to observe the progressive fatality arising from delay. Suffering above 30 hours destroys one in 34; in other 10 hours the danger more than doubles, for 1 in 13 die; then 1 in 11, and next 1 in 8, to say nothing of the children." 423-4.

Upon the whole, we think, that Dr. Burns has put in a very proper point of view the theory and practice of instrumental aid. It has fallen to our lot on various occasions to regret, that recourse to the forceps had not been more early sought. We have witnessed several instances in which the life of the child has been lost, for want of seasonable assistance from these instruments, and sometimes the mother has been placed in a very unwarrantable state of hazard from the delay. While therefore we join heart and hand with those who deprecate the hasty and intemperate use of instruments, we as earnestly insist upon the propriety of having timely recourse to them, when the circumstances of the labour indicate a want of power in Nature, to discharge her duty satisfactorily.

The instructions for introducing, and acting with, the forceps, are sufficiently clear and intelligible. Dr. Burns seems not anxious to recommend any particular form of forceps. It is with this as with other instruments, the more simple, generally the more manageable. Dr. Burns allows the long forceps to be introduced upon some occasions, but very judiciously cautions practitioners not to be too confident with them. In our opinion it is a very hazardous instrument. In a *narrow* pelvis, we have never known it save the life of the child, and we do know some cases, in which it occasioned extreme suffering and distress to the mother.

But reflecting that we are reviewing the *fifth* edition of an elementary work, we find our limits considerably exceeded.

Dr. Burns cannot fail to impress every reader with a high respect for his talents, his judgment, his unwearied industry —and last, not least, his ardent zeal for the promotion and diffusion of obstetric science. The volume is a real treasure to the general practitioner, as containing an almost inexhaustible store of the most valuable and concentrated information. Well may our author conclude his preface in the following terms.

"I do, however, sincerely trust, that the precepts I have inculcated will be found agreeable to experience;—and, on a review of the whole, I cannot say that I have either wasted the reader's time in idle theory, or misled his opinion by mere speculation."

The whole work has been carefully revised, and the additions to this impression exceed one hundred pages.

